# The Frequency and Determinants of Liver Stiffness Measurement Failure: A Retrospective Study of “Real-Life” 38,464 Examinations

**DOI:** 10.1371/journal.pone.0105183

**Published:** 2014-08-14

**Authors:** Dong Ji, Qing Shao, Ping Han, Fan Li, Bing Li, Hong Zang, Xiaoxia Niu, Zhongbin Li, Shaojie Xin, Guofeng Chen

**Affiliations:** 1 Liver Fibrosis Diagnosis and Treatment Center, 302 Military Hospital of China, Beijing, China; 2 Tumor Radiotherapy Center, 302 Military Hospital of China, Beijing, China; 3 Liver Failure Diagnosis and Treatment Center, 302 Military Hospital of China, Beijing, China; Yonsei University College of Medicine, Republic of Korea

## Abstract

**Objective:**

To investigate the frequency and determinants of liver stiffness measurement (LSM) failure by means of FibroScan in “real-life” Chinese patients.

**Methods:**

A total of 38,464 “real-life” Chinese patients in 302 military hospital of China through the whole year of 2013, including asymptomatic carrier, chronic hepatitis B, chronic hepatitis C, liver cirrhosis (LC), alcoholic liver disease, autoimmune liver disease, hepatocellular carcinoma (HCC) and other, were enrolled, their clinical and biological parameters were retrospectively investigated. Liver fibrosis was evaluated by FibroScan detection. S probe (for children with height less than 1.20 m) and M probe (for adults) were used. LSM failure defined as zero valid shots (unsuccessful LSM), or the ratio of the interquartile range to the median of 10 measurements (IQR/M) greater than 0.30 plus median LSM greater or equal to 7.1 kPa (unreliable LSM).

**Results:**

LSM failure occurred in 3.34% of all examinations (1286 patients out of 38,464), among them, there were 958 cases (2.49%) with unsuccessful LSM, and 328 patients (0.85%) with unreliable LSM. Statistical analyses showed that LSM failure was independently associated with body mass index (BMI) greater than 30 kg/m^2^, female sex, age greater than 50 years, intercostal spaces (IS) less than 9 mm, decompensated liver cirrhosis and HCC patients. There were no significant differences among other diseases. By changing another skilled operator, success was achieved on 301 cases out of 1286, which reduced the failure rate to 2.56%, the decrease was significant (*P*<0.0001).

**Conclusions:**

The principal reasons of LSM failure are ascites, obesity and narrow of IS. The failure rates of HCC, decompensated LC, elder or female patients are higher. These results emphasize the need for adequate operator training, technological improvements and optimal criteria for specific patient subpopulations.

## Introduction

Formation and accumulation of fibrosis in the liver is the common pathway of chronic liver diseases (CLD) that represent a major cause of morbidity and mortality worldwide. An accurate assessment of the extent of liver fibrosis is important for predicting the prognosis and determining the appropriate management of patients with CLD [1]–[4]. Until recently, liver biopsy (LB) is still considered as the best way for this purpose, and remains the gold standard in assessing liver histology. Although percutaneous LB is a safe procedure, it is an invasive one with rare but potentially life-threatening complications, resulting which can lead to treatment delay. Ultimately, its accuracy has been questioned because of sampling errors and inter- or intra-observer variability. So, noninvasive techniques have developed recently and become more popular every day due to better patient compliance. Liver stiffness measurement (LSM) using FibroScan (Echosens, Paris, France) is one of these novel noninvasive methods for assessing liver fibrosis and therefore cirrhosis, both in chronic hepatitis C and other CLD [5]–[9]. A LSM was considered reliable if the ratio of the interquartile range to the median of 10 measurements (IQR/M) less than 0.3, or IQR/M >0.30 with median liver stiffness <7.1 kPa. The diagnostic accuracy of FibroScan is excellent for cirrhosis and mild fibrosis. Because FibroScan is a user-friendly technique that can be performed rapidly at bedside, with immediate results and high acceptance of patients and physicians, it is likely to become an important tool in clinical practice in the near future [10]–[15].

However, FibroScan has its own limitation: its results may be influenced by high alanine aminotransferase (ALT) level and extrahepatic cholestasis. In addition, FibroScan is difficult in patients with obesity, narrow intercostal spaces, or ascites [16]–[19]. In this study, we retrospectively investigated the frequency and determinants of LSM failure in “real-life” Chinese patients, based on 38,464 examinations.

## Patients and Methods

### Patients

The cohort study included 38,464 “real-life” patients with CLD due to various causes, who had undergone FibroScan detection at 302 military hospital of China, Beijing, China, through a whole year of 2013. Hepatitis B (HBV) or C (HCV) virus infection was diagnosed by serological detection of HBV surface antigen and HCV antibody, and was based on 2012 Asian-Pacific consensus statement on the management of chronic hepatitis B issued by Asian Pacific Association for the study of the liver and 2011 European HCV clinical practice guidelines issued by European association for the study of the liver [20], [21], respectively. Alcoholic liver disease (ALD) was diagnosed in patients with consumption of at least 40 g alcohol daily for at least 5 years. The diagnosis of hepatocellular carcinoma (HCC) was established using combination of elevated AFP and typical findings on tri-phasic CT or MRI. Other diseases were diagnosed according to the current criteria. LSMs are median values of successful acquisitions. The following clinical and biological parameters were recorded at the time of LSM: age, sex, body mass index (BMI), intercostal space (IS), and ALT.

The written informed consents were obtained from all patients for their clinical records to be used in this study, and their information was anonymized and de-identified prior to analysis. The study was approved by ethics committee of 302 military hospital of China and was conducted in accordance with the ethical standards formulated in the Helsinki Declaration.

### Liver stiffness measurement

Liver stiffness was measured using FibroScan device (Echosens, Paris, France) according to the manufacturer’s instructions. Measurements were performed on the right lobe of the liver through the IS with patients lying in a dorsal decubitus with the right arm in maximal abduction. S probe (for children with height less than 1.20 m) and M probe (for adults) were used. The tip of the transducer probe was covered with coupling gel and placed on the skin, between the ribs at the level of the right lobe of the liver. The operator, assisted by a time-motion ultrasound image, located a liver portion that was at least 6-cm thick and free of large vascular structures. When the target area had been located, the operator pressed the probe button to commence the measurements. Ten validated measurements were performed on each patient. The results were expressed in kilopascals (kPa), with a higher value reflecting a stiffer liver and more severe liver fibrosis. The results were considered failure when no value was obtained after at least 10 shots (unsuccessful LSM), or when the IQR/M greater than 0.30 plus median LSM greater or equal to 7.1 kPa (unreliable LSM). If a patient had a LSM failure, the second LSM examination was performed with another skilled operator at least 2 days, but no longer than 1 week after first examination.

### Statistical analysis

All statistical analyses were performed using the SPSS version 19.0 (SPSS Inc., Chicago, IL, USA). In descriptive analyses, continuous variables were expressed as mean ± standard deviation and categorical variables as absolute figures and percentages. Chi-squared test was used for categorical variables and Fisher’s exact test when appropriate. Continuous variables with skewed distribution were analyzed using Mann-Whitney test. Factors significantly associated with the outcome in univariate analyses were entered in a multivariate logistic model. Odd ratios were estimated from the model and are given with their 95% confidence intervals (CI). A *P*-value of <0.05 was considered significant.

## Results

### Patient characteristics

A total of 38,464 examinations were performed, mean age was 43.5±12.2 years (from 11 to 90 years), 13112 (34.1%) of patient was older than 50 years, 9541 (24.8%) were females, mean BMI was 24.2±3.4 kg/m^2^, 3536 (9.2%) were obesity (BMI≥30 kg/m^2^). The median (IQR) of LSM value and ALT were 7.6 (5.5–12.3) kPa and 39 (23–89) IU/ml. The mean examination duration was 4 min and 35 seconds.

**Figure 1:**
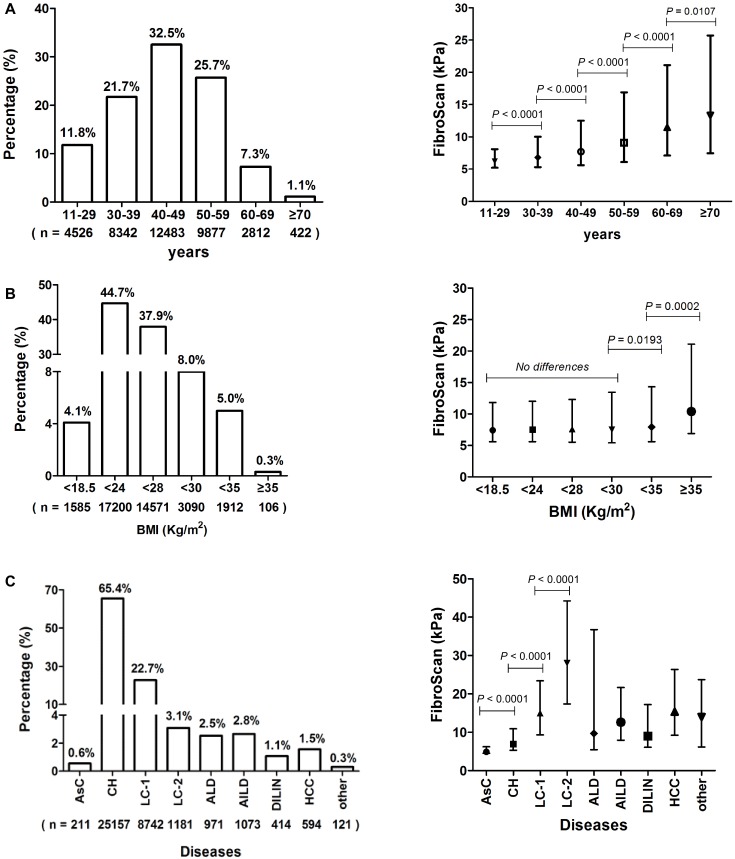
Summary of 38,464 LSM examinations according to age, BMI, and diseases group. LSM values are presented as median (interquartile range). Nonparametric test **(**Mann-Whitney test) is used to analyze the differences between each two groups. Patients’ percentage and FibroScan value distribution are analyzed according to age (A), BMI (B), and diseases (C) group. AsC, chronic asymptomatic HBV carrier; CH, chronic hepatitis; LC-1, compensated liver cirrhosis; LC-2, decompensated liver cirrhosis; ALD, alcoholic liver disease; AILD, autoimmune liver disease; DILIN, drug-induced liver injure; HCC, hepatocellular carcinoma (HCC); other includes liver transplant recipient, hepatolenticular degeneration, and Budd-chiari syndrome.

The distribution of enrolled patients grouped by years was almost normal one, the percentage of patients with 40–49 years old was the highest (32.5%), and there were significant differences on FibroScan values according to age groups (Figure 1A).

Based on BMI, there were no differences among patients with BMI<30 kg/m^2^, however, for those patients with BMI≥30 kg/m^2^, LSM value raised with their BMI increasing significantly (Figure 1B).

Indications for LSM by means of FibroScan were chronic asymptomatic HBV carrier (AsC) 0.6%, chronic viral hepatitis (CH) 65.4%, compensated liver cirrhosis related to viral hepatitis (LC-1) 22.7%, decompensated LC related to viral hepatitis (LC-2) 3.1%, alcoholic liver disease (ALD) 2.5%, autoimmune liver disease (AILD) 2.8%, drug-induced liver injure (DILIN) 1.1%, hepatocellular carcinoma (HCC) 1.5%, and other diseases (including liver Transplant Recipient, hepatolenticular degeneration, and Budd-chiari syndrome) 0.3%. With the progress of chronic viral hepatitis, the LSM value increased significantly (P<0.0001) (Figure 1C).

### Frequency of LSM Failure in the total population

**Figure 2:**
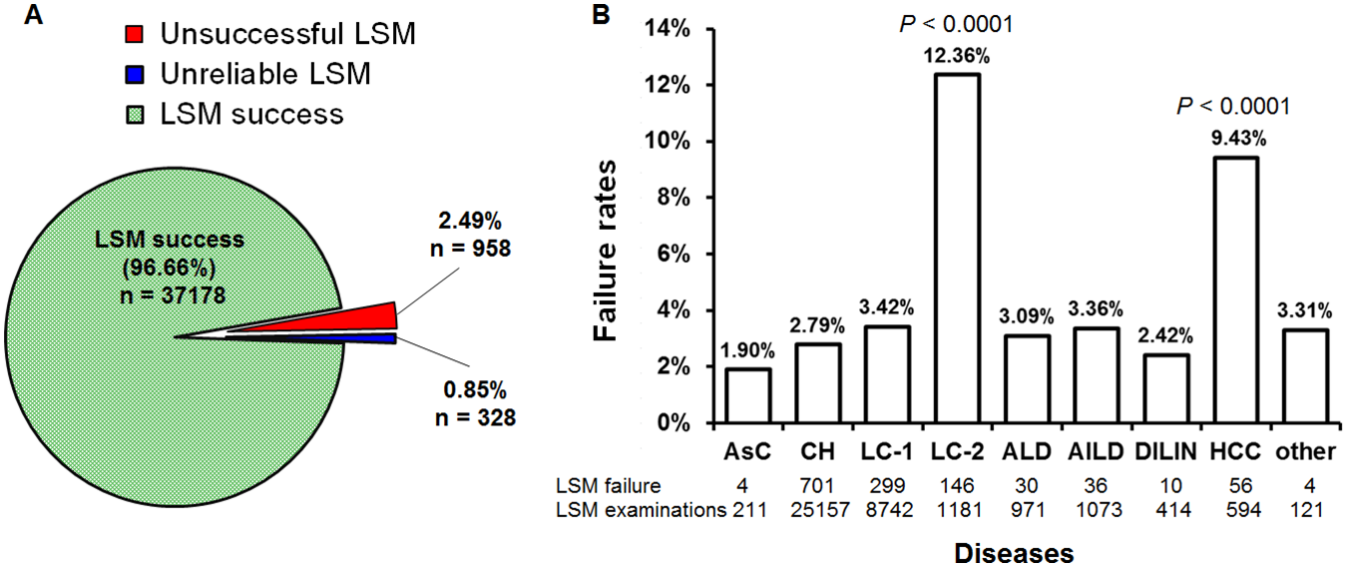
Frequency of LSM failure according to different diseases. LSM failure rate in 38,464 LSM examinations is overviewed (A), and Chi-square test is applied to find the unsuccessful and unreliable LSM rate differences between each disease group and the total failure rate (B). AsC, chronic asymptomatic HBV carrier; CH, chronic hepatitis; LC-1, compensated liver cirrhosis; LC-2, decompensated liver cirrhosis; ALD, alcoholic liver disease; AILD, autoimmune liver disease; DILIN, drug-induced liver injure; HCC, hepatocellular carcinoma (HCC); other includes liver transplant recipient, hepatolenticular degeneration, and Budd-chiari syndrome.

Overall, LSM failed in 1286 patients (3.34%), which included 958 cases (2.5%) with unsuccessful LSM, and 328 cases (0.9%) with unreliable LSM (Figure 2A). Mean age of these 1286 patients was 51.1±9.5 years, 869(67.6%) were females. LSM values were obtained in 37178 (96.66%) patients. LSM failure ranged from 1.90% in AsC patients to 12.36% in patient with decompensated LC (most of them with obvious ascites), Chi-squared test showed that LSM failure rate was statistical significant high in decompensated LC and HCC patients (*P*<0.0001) compared with the total failure rate (3.34%) (Figure 2B). On second examination (at least two days, but no longer than one week after first examination) by changing another skilled operator, the fail rate reduced to 2.56% (985 cases).

### Factors associated with LSM failure

**Figure 3:**
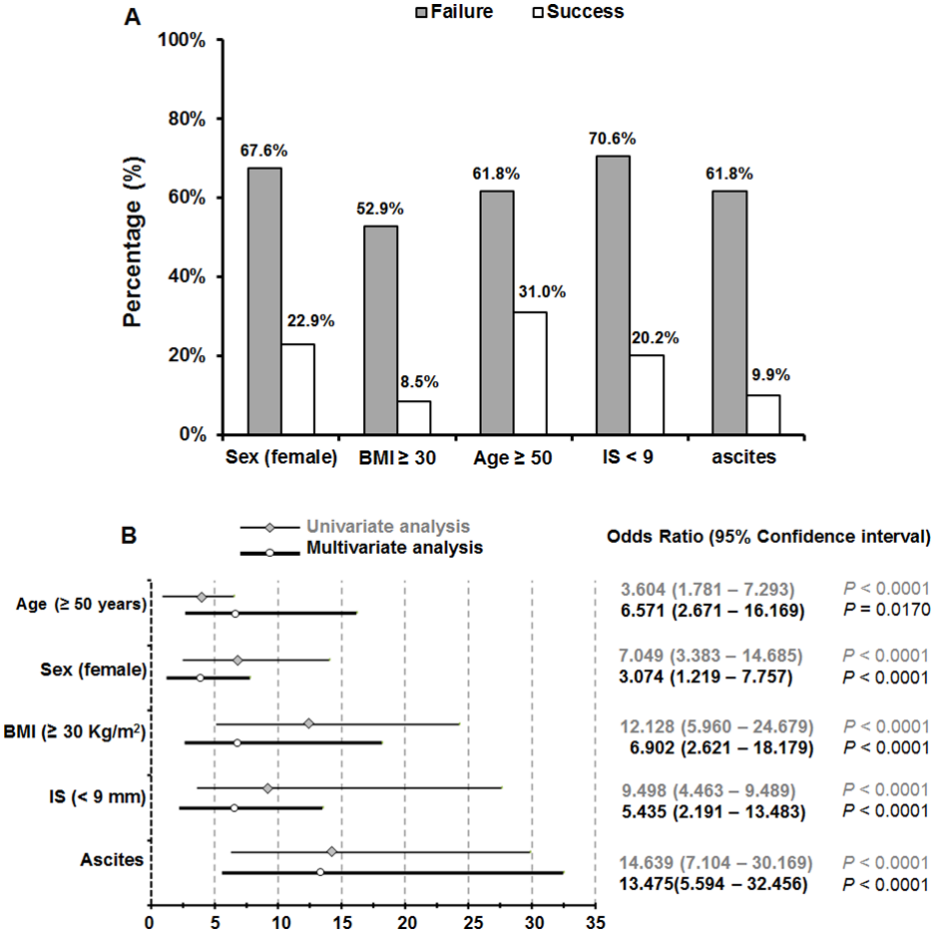
Distribution of factors significantly associated with LSM failure. Patients’ percentage distribution according to the factors associated with LSM failure is showed (A), and the significances were determined by univariate and multivariate analyses (B). Intercostal space (IS).

Altogether five factors showed significant association with LSM failure, the distribution of these 5 factors is presented in Figure 3A. Multivariate analysis showed that LSM failure was independently associated with the following factors: ascites (OR 13.475, 95% CI 5.594–32.456), BMI greater than 30 kg/m^2^ (OR 6.902, 95% CI 2.632–18.179), age older than 50 years (OR 6.571, 95% CI 2.671–16.169), IS narrower than 9 mm (OR 5.435, 95% CI 2.191–13.483), and female (OR 3.074, 95% CI 1.219–7.757). Univariate analysis had the similar results (Figure 3B).

## Discussion

Hepatic fibrosis is the final result of CLD and is a wound healing process similar to those observed in other organs. So, early diagnosis of fibrosis or cirrhosis is an important clinical issue, as it is a pivotal factor in determining a treatment plan for antiviral therapy and predicting the overcome of CLD [22]–[24]. LSM was first studied in Western populations with chronic hepatitis C. Recently, it has been used in the assessment of other hepatic fibrosis-related non-HCV etiologies such as HBV, ALD, AILD and non-alcoholic hepatitis [25]–. All of these studies have shown that LSM values correlate strongly with METAVIR fibrosis staging by LB. LSM is measured through a device that is called FibroScan which is composed of an ultrasound transducer probe mounted on the axis of a vibrator. Vibrations of mild amplitude and low frequency are transmitted by the transducer, inducing an elastic shear wave that propagates through the underlying tissues. Pulse echo ultrasound acquisition is used to follow the propagation of the shear wave and to measure its velocity, which is directly related to tissue stiffness: the stiffer the tissue, the faster the shear wave propagates. FibroScan measures liver stiffness in a volume that is approximately a cylinder 1 cm wide and 4 cm long, between 2.5 cm and 6.5 cm below the skin surface. This volume is at least 100 times bigger than a biopsy sample. FibroScan examination is painless, rapid (less than 5 min) method with a steep learning curve and requires the experience of only 50–100 examinations to be able to make proficient determinations, which is easy to perform at the bedside or in the outpatient clinic. In various studies, the accuracy of FibroScan results was similar to that of serum non-invasive markers for the diagnosis of significant fibrosis, sometimes with inadequate figures (<80%), and showed excellent performance for the diagnosis of cirrhosis.

Over the last few years, LSM has been increasingly used as a noninvasive measurement for the assessment of liver fibrosis and even adopted as first-line screening tool for patients with CLD. Although the usefulness of LSM has been established, there is relatively limited data on its failure. The current study is to clarify the reason of LSM failure and comprehend the factors contributed to LSM failure. LSM can be difficult in obese patients or in those with narrow IS and impossible in patients with ascites. Failure rates range between 2.4% and 9.4% in the different studies [29]–[32].

In the present study, we could not obtain 1286 valid measurements in 38,464 examinations, which led to a failure rate of 3.34%. Among them, there were 958 cases (2.5%) with unsuccessful LSM, and 328 cases (0.9%) with unreliable LSM. These data were similar as to the previous study which also performed in Chinese patients [33]. A significant decrease in the failure rate was observed in patients undergoing second examination (3.34% at 1^st^ versus 2.56% at 2^nd^ examination, *P*<0.0001). To address the reasons for a higher successful LSM on 2nd examination, we thought that the experience of operators (e.g. probe positioning, probe pressure) might be a major factor, but patients body position, respiratory movements or even patients’ psychological state also could influence the results of LSM, the impacts of these factors need more researches to be clarified.

Regarding the etiologies of CLD, we found that the failure rates in decompensated LC and HCC patients were significant higher than other diseases, which might be associated with ascites complication in decompensated LC patients and uneven distribution of liver parenchyma of HCC patients. We also found that LSM is more difficult in patients with IS narrower than 9 mm compared with those normal IS patients. These results suggest that operator experience might be important to gain the valid results, and operators should be fully trained before work in clinic.

Another important determinant of LSM failure was obesity (BMI≥30 kg/m^2^). Our study showed that 52.9% of LSM failure was patient with BMI more than 30 kg/m^2^, while only 8.5% in LSM success. The reason we supposed that obesity patients are large waist circumference, and the subcutaneous and prehepatic fat thickness is increased, attenuating both elastic waves and ultrasound and making LSM impossible with a regular probe that is calibrated for a given distance between the liver and the chest wall. Recently, the manufacturer of transient elastography developed a new XL probe for obese patients, it is possible to generate low-frequency ultrasound to evaluate deeper liver tissue. A study showed the success rate of measurement by XL probe can be as high as 90% even in obese patients [34]. Another study of 193 consecutive nonalcoholic fatty liver disease (NAFLD) patients (35% patients with BMI≥30 kg/m^2^) showed that XL probe was more likely to achieve 10 valid measurements compared with M probe (95% vs. 81%; *P*<0.001) in these patients, and among patients with BMI≥30 kg/m^2^, 93% had successful LSM using XL probe, compared with only 60% when M probe was used (*P*<0.0001). The study also emphasized that because validation data on XL probe are less extensive than those on M probe (XL probe tends to generate higher IQR/M), M probe should be used in all patients as first line. In the patients who failed M probe measurements, XL probe could be used as a salvage technique to ensure higher successful measurements in most obese patients [35]. The other important point is that LSM values obtained with XL probe were typically 1.0–2.0 kPa lower than those obtained with M probe [36], [37], So in order to improve the diagnosis accuracy of XL probe for obese patients, more clinical researches should be performed to set up the optimal criteria and standard procedure of XL probe examination.

LSM failure rate is relatively high in patients with HCC and decompensated LC, IS narrower than 9 mm, BMI more than 30 kg/m^2^, female sex, and age older than 50 years, these results emphasize the need for adequate operator training, technological improvements, and optimal protocol establishment in specific patient subpopulations.

In conclusion, LSM by means of FibroScan is a promising, reproducible noninvasive technique of evaluation which could be used in numerous clinical situations, and efforts should be made to avoid examination failure and set up the standard of its practice.
